# Receipt of long-acting injectable antiretroviral therapy among people with HIV in Southern US states: an assessment using electronic health records and claims data

**DOI:** 10.1186/s12981-024-00690-9

**Published:** 2025-02-01

**Authors:** Yiyang Liu, Rebecca J. Fisk-Hoffman, Maitri Patel, Robert L. Cook, Mattia Prosperi

**Affiliations:** Department of Epidemiology, College of Public Health and Health Professions and College of Medicine, University of Florida, Gainesville, FL US

**Keywords:** HIV, Long-acting antiretroviral therapy, Electronic health records, Cabotegravir/Rilpivirine

## Abstract

**Background:**

In January 2021, the United States (US) Food and Drug Administration (FDA) approved the first long-acting injectable antiretroviral therapy (LAI ART) regimen for the treatment of HIV providing an alternative to daily oral regimens. We analyzed electronic health records (EHRs) to provide real-world evidence of demographic and clinical characteristics associated with the receipt of LAI ART among people with HIV (PWH).

**Methods:**

Leveraging EHRs from a large clinical research network in the Southern US - OneFlorida + linked with Medicaid (updated to 08/2022) - we identified a cohort of PWH who have been prescribed at least one dose of LAI ART since January 2021 and characterized their demographics, clinical characteristics, and HIV care outcomes.

**Results:**

A total of 233 LAI ART recipients were identified: 56.7% female, 45.1% aged 30 to 44, 51.3% non-Hispanic Black, 78.1% on Medicaid and 4.7% on private insurance. Approximately three-quarters of injections (71.2%) were received within 37 days of the previous dose, and 84.4% were received within 67 days. About 8% of LAI ART recipients did not have optimal care engagement the year before LAI ART initiation; one in five recipients had a diagnosis of alcohol or substance use disorder in lifetime. All achieved viral suppression (< 50 copies/mL) before starting LAI ART. Of a subset of patients with HIV viral load test records, only 1 record of virologic failure (viral load > 200 copies/ml) was observed after the initiation of LAI ART.

**Discussion:**

There has been an increasing trend of LAI ART initiation since approval. People with suboptimal care engagement and with substance use disorder in lifetime were not excluded from LAI ART treatment.

**Supplementary Information:**

The online version contains supplementary material available at 10.1186/s12981-024-00690-9.

## Introduction

Although there is no cure for HIV currently, adherence to antiretroviral therapy (ART) allows for people with HIV (PWH) to achieve viral suppression, which significantly decreases rates of mortality and morbidity, increases quality of life, and prevents transmission (Undetectable = Untransmittable, U = U) [1, 2]. In the United States (US), the widespread availability and success of ART have led to a notable shift in the landscape of HIV care, including aiding more PWH achieve viral suppression in a shorter period of time [3]. The creation of effective daily multi-drug, single-tablet regimens simplified the administration of the multiple drug regimens required to effectively suppress HIV replication, increasing adherence and the odds of virologic success [4]. Despite the simplified regimens, many PWH still face barriers to adherence, undermining the effectiveness of ART [5]. Suboptimal adherence to daily ART has myriad causes including distress from the daily reminder of HIV, concerns about inadvertent disclosure, trouble swallowing, concerns over drug-drug interactions, overall medication burden, and simply forgetting due to changes to their schedule or busy lives [6, 7]. These barriers increase the risk of virologic failure, the development of drug resistance, and increased HIV transmission. Further, barriers to adherence and negative HIV-related health outcomes disproportionately effect PWH with lower socio-economic status, younger PWH, PWH who identify as non-Hispanic Black or Hispanic, and PWH with substance use disorders [8–11]. Therefore, there has been increasing interest in the potential benefits of long-acting injectable (LAI) ART across a variety of patient demographics [12].

Multiple clinical trials [12–15] established the safety and efficacy of LAI ART using Cabotegravir (CAB) and Rilpivirine (RPV), leading to the US Food and Drug Administration (FDA) approval in January 2021. The LAI CAB/RPV, administered either monthly or bi-monthly after an initial monthly dose, has been shown to sustain viral suppression in treatment experienced PWH [16]. The strict eligibility criteria for the clinical trials have influenced the prescribing guidelines (i.e., requiring sustained viral suppression before initiating) and may limit the generalizability of the results to the broader population of PWH. Preliminary data and observational studies have indicated that PWH who have previously had detectable viral loads due to adherence barriers have been able to achieve and sustain viral suppression on LAI CAB/RPV, although it is unclear how often it is being used off label in the real world [17–20]. Additionally, providers may be hesitant or unable to prescribe LAI CAB/RPV due to the patient’s body size, access to transportation, current insurance provider, and reliability in keeping HIV appointments [21–24]. Despite these concerns and barriers, LAI CAB/RPV offers a treatment option that could help many PWH achieve viral suppression and overcome disparities in HIV-related outcomes.

In 2019, the US announced the Ending the HIV Epidemic Initiative (EHE) which aims to reduce the number of new HIV infections by 90% by 2023, and seven counties in Florida were selected as EHE priority jurisdictions: Broward (Fort Lauderdale), Duval (Jacksonville), Hillsborough (Tampa), Miami-Dade, Orange (Orlando), Palm Beach, and Pinellas. Beyond the priority counties, Florida is a high HIV prevalence and incidence setting, with a significant burden of HIV in rural areas. Georgia and Alabama also have high HIV incidence regions that are targeted by the EHE. Therefore, it is important to understand early access to LAI CAB/RPV to help identify populations and areas where expanding access may better help the state meet the EHE goals. This includes understanding whether initiation differs by factors associated with disparities in HIV-related outcomes. In the present study, we used real-world data, comprising of electronic health records (EHRs) and linked Medicaid claims data, to identify a cohort of PWH who received LAI ART, and compared their demographic and clinical characteristics with PWH who received oral ART during the same period.

## Methods

### Data source

We used data from OneFlorida+, one of the eight Clinical Research Networks funded by the Patient-Centered Outcomes Research Institute (PCORI). OneFlorida includes longitudinal EHRs from 14 healthcare systems dating back to January 2012, linked to Medicaid claims (both medical and pharmacy); all are sourced via a common data model and stored in a centralized repository. As of 2022, OneFlorida + included over 17 million patients from Florida, 2.1 million from Georgia, and 1.1 million from Alabama. For the present analyses, we accessed a de-identified database from all OneFlorida sites, updated to August 2022. The study protocol was approved by the University of Florida’s Institutional Review Board (#IRB202002581). For study replication purposes, data requests can be made to the OneFlorida consortium: https://onefloridaconsortium.org/front-door/research-infrastructure-utilization-application, and the authors can provide full documentation on the data extraction procedures.

### Study sample

A cohort of PWH was identified using a previously validated computable phenotype (98.9% sensitivity and 97.6% specificity) [25]. In brief, the algorithm identifies PWH based on HIV diagnosis codes along with either positive HIV RNA or Ag/Ab results, records of ART medications, or three or more clinical encounters where HIV diagnosis codes are present. To assess representativeness of the Florida portion of our sample, we compared the most common ZIP codes (first three digits) of patients’ residential addresses in our sample with Florida Department of Health estimates [26]. We found that the areas from which most PWH in our sample reside align well with the department’s estimates of high HIV prevalence, with a slight over-representation of patients from Duval County/City of Jacksonville (data not shown).

People who received LAI CAB/RPV were identified by screening a combination of medication names, RxNorm, and National Drug Code from prescribing and dispensing records. We included all patients who received prescriptions for at least one dose of LAI CAB/RPV after January 2021, the FDA approval date for this treatment regimen. Five individuals received LAI CAB/RPV in 2019, before the FDA approval date and were likely to be clinical trial participants. Among them, four also received LA CAB/RPV after January 2021 but the one person to whom LAI CAB/RPV was only administered in 2019 was excluded from the current analyses. Additionally, we identified individuals who received oral ART during the same period (i.e., after January 2021). For LAI ART recipients, we established the index date as the date when they initiated LAI ART. For oral ART recipients, the index date was set as January 1, 2021.

### Data elements

#### Socio-demographics

We considered age, self-reported sex, race, and ethnicity. Age was calculated as of the index date and grouped into 18 to 29, 30 to 44, 45 to 64, or 65 or older. Race and ethnicity were grouped into Hispanic, non-Hispanic White, non-Hispanic Black, non-Hispanic other, or unknown. Primary payer was categorized into Medicaid, Medicare, private insurance, no insurance/self-pay, other governmental insurance, unspecified other/unknown. Individuals at health risk due to socioeconomic and psychosocial factors were inferred from EHRs and claims by considering the Z codes (Z55 to Z65) from the International Classification of Diseases, Tenth Revision (ICD-10). The first three digits of patients’ residential ZIP codes were extracted.

#### Clinical characteristics

Optimal engagement HIV care was defined as having at least two HIV-related care visits (including an HIV diagnosis code, ART prescription, or recorded HIV labs during a clinical encounter) with at least a 90-day gap in a year [27]. Viral suppression and virologic failure were estimated for a subsample of individuals with available HIV laboratory results (*n* = 53), as not all patients had laboratory test records (e.g., those identified from claims lacked laboratory records). HIV viral suppression was defined as a HIV RNA load < 50 copies/mL at the most recent HIV-related visit in a calendar year. Virologic failure was defined as a single viral load > 200 copies/mL at any time on LAI CAB/RPV.

Lifetime diagnoses of substance use disorder were identified using both ICD Ninth Revision (ICD-9) and ICD-10 codes and included: tobacco use disorder, alcohol use disorder, cannabis use disorder, cocaine use disorder, opioid use disorder, amphetamine use disorder, and sedative use disorder. All the ICD codes utilized have been included in the appendix and were selected based on a prior study [28] and are following the criteria outlined in the Diagnostic and Statistical Manual of Mental Disorders, Fifth Edition (DSM-5) [29].

Chronic comorbidity burden was assessed using the Charlson comorbidity index (CCI) [30, 31]. We calculated a CCI score, excluding HIV/AIDS, which is originally included in the CCI, as our sample consists of PWH. Having a CCI score of zero indicates the absence of comorbidity, while a score of 1 or 2 indicates mild comorbidity, 3 or 4 indicates moderate comorbidity, and a score greater than or equal to 5 indicates severe comorbidity [32].

We extracted oral ART medication prescriptions and dispensing records for the year before the index date. For individuals with at least one ART record during that year, we categorized them based on whether they received any Non-Nucleoside Reverse Transcriptase Inhibitors (NNRTIs), Nucleoside Reverse Transcriptase Inhibitors (NRTIs), Protease Inhibitors (PIs), or Integrase Strand Transfer Inhibitors (INSTIs). Additionally, we examined their oral ART regimens, identifying the top four most common patterns: NRTI + INSTI, NRTI + PI, NRTI + INSTI + PI, and NRTI + NNRTI. All other ART patterns were grouped into “other,” resulting in five mutually exclusive patterns to represent the ART regimens. Among people who initiated LAI CAB/RPV, we calculated the total number of LAI CAB/RPV prescription and dispensing records, assessed the HIV treatment profile after LAI CAB/RPV initiation, and measured the length of time between LAI CAB/RPV prescription/dispensing records. We classified HIV treatment following the initiation of LAI CAB/RPV based on the timing and type of ART records: only received LAI CAB/RPV (no oral ART records after LAI initiation), concurrent oral ART and LAI CAB/RPV use (receiving oral ART during periods of LAI CAB/RPV), and continuation with oral ART (restarted oral pills at least 67 days after last LAI CAB/RPV records).

### Analysis

Data processing and analyses were carried out using SAS 9.4. We performed stratified descriptive statistics and tested for differences between LAI CAB/RPV and oral ART recipients using the Mantel-Haenszel Chi-Square test. Furthermore, we identified the month and year when a patient first initiated LAI CAB/RPV, calculated cumulative rate of LAI initiation with the total number of PWH with clinical encounters in 2021 and 2022 as the denominator, and plotted the trend of the number of patients who began LAI CAB/RPV between January 2021 and August 2022.

## Results

A total of 87,256 PWH were identified from OneFlorida + data since 2012, and 22,834 PWH had clinical encounters and ART prescription or dispensing records in 2021 and 2022 comprised the study sample. Among them, 233 received any LAI CAB/RPV prescription and 22,611 had only been prescribed oral ART.

**Fig. 1:**
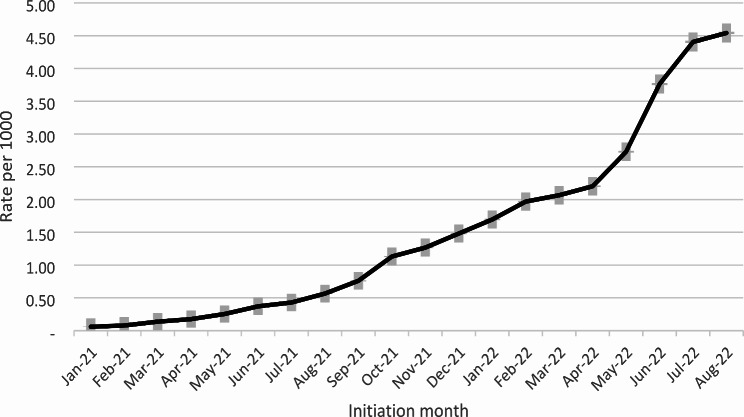
Trends in LAI initiation rate (cumulative) after FDA approval date Denominator: total number of PWH who had clinical encounter between 2021 and 2022 Grey bar represents the 95% confidence interval

Overall, an increasing trend of LAI CAB/RPV initiation has been observed since the FDA approval date in Jan 2021 (Fig. 1). As shown in Table 1, LAI CAB/RPV recipients were more likely to be female (56.7% vs. 43.0%), aged 30–44 (45.1% vs. 30.1%), and on Medicaid (78.1% vs. 62.9%). The distribution of racial/ethnic groups did not notably differ by LAI CAB/RPV initiation status.

Around 8% of LAI CAB/RPV recipients did not have documented optimal HIV care engagement one year before the index date, lower than the 13.4% observed among oral ART recipients. Additionally, oral ART recipients had a much higher proportion of having no recorded HIV care encounters in the year before the index date (i.e., no data; 5.6% vs. 37.2%) when compared with LAI CAB/RPV recipients. Among a subset of people who had viral load records, all people who initiated LAI CAB/RPV achieved HIV viral suppression before the index date, while only 24.3% oral ART recipients had achieved the same. NRTIs were the most commonly prescribed regimens in the year before the index date, though less frequent among LAI CAB/RPV recipients than oral ART users (86.8% vs. 93.0%). LAI CAB/RPV recipients were also more likely to be on NNRTI regimens (25.4% vs. 14.6%). NRTI + INSTI was the most common ART regimen pattern for both LAI and oral ART recipients (59.0% and 63.0%).

A consistently low proportion of individuals at health risk due to socioeconomic and psychosocial factors was observed among LAI and oral ART recipients (6.4% vs. 6.9%). Additionally, a lower proportion of LAI CAB/RPV recipients had alcohol (14.6% vs. 19.8%) and amphetamine (3% vs. 6.3%) use disorders. No notable differences were observed for other substance use disorders and the comorbidity score between LAI and oral ART recipients.

Detailed clinical characteristics of the identified LAI CAB/RPV recipients are presented in Table 2. More than half of the LAI CAB/RPV recipients had 2–4 prescribing/dispensing records of LAI CAB/RPV, 13.3% had 5–9 records, and 3.9% had 10 or more records. Of all LAI CAB/RPV records, 56.7% were for the 3 mL kit (CAB 600 mg/RPV 900 mg), 39.5% were for the 2 mL kit (CAB 400 mg/RPV 600 mg), and 4.4% did not specify the dose (data not shown). LAI CAB/RPV is considered a complete HIV regimen and 54.1% of LAI CAB/RPV recipients have only been prescribed LAI CAB/RPV, 43.8% concurrently received other oral ART, and 2.2% continued with oral ART. Among people who had at least 2 LAI CAB/RPV records (*n* = 159), the average times between each prescribing/dispensing record were 50.7 days (SD = 45.3); the median time was 28 days and did not vary by dosing regimen. Most (71.2%) injections were documented within 37 days (one month plus one-week flexible window) from the previous records, and 84.4% within 67 days (two months plus one-week flexible window, data not shown). However, 42.3% of the people experienced at least one time gap between LAI CAB/RPV records that exceeded 67 days. Of the 68 PWH with a longer gap, 47.1% were prescribed both oral ART and LAI CAB/RPV, and these individuals were more likely to have an unknown insurance status and only have EHR data (data not shown). No clear oral bridging patterns were observed (i.e., the oral pills observed during LAI were not oral CAB/RPV). Among the 53 PWH with available viral load data, only one person experienced virologic failure after LAI initiation. The areas where LAI CAB/RPV prescription was predominantly observed, based on the first three digits of patients’ residential ZIP codes, are Duval, Miami-Dade, Palm Beach, and Pinellas counties.

## Discussion

This study found differences in LAI CAB/RPV initiation by sex, age, type of insurance, documentation of an alcohol use disorder, care engagement, and viral suppression prior to the index date. Among those prescribed LAI CAB/RPV, around half were only on LAI CAB/RPV without concurrent prescriptions of an oral ART, and a minority switched back to oral ART within the study period. We found that virological failure on LAI CAB/RPV was rare. We also observed around half of all LAI CAB/RPV initiators with multiple records having gaps between injections greater than the recommended guidelines.

Compared with other EHR analyses, we found a higher proportion of patients receiving LAI CAB/RPV identified as females (56.7% vs. 16% found in OPERA cohort) and Black (51.1% vs. 37%) [33]. Additionally, we observed that females and individuals aged 30–44 were more represented among LAI CAB/RPV recipients than among oral ART recipients. This differed from a past study of patient preferences for analogs to the existing LAI CAB/RPV, where men were more likely to prefer a long-acting option [34]. In contrast, the higher initiation rates of LAI CAB/RPV among younger populations aligned with the findings from a previous study [34]. Notably, we did not find strong differences in initiation by race/ethnicity, which did not align with past studies on preference and differed from the rollout of highly active ART in the early 2000’s which increased racial and ethnic disparities in HIV-related health outcomes due to differing access to these new medications [34–38]. A low proportion of individuals had diagnosis codes indicating health risk from socioeconomic and psychosocial factors, likely due to the underutilization of these codes for documenting social determinants of health in EHRs [39]. The majority of LAI CAB/RPV recipients resided in counties with high HIV burden (including Miami-Dade, Palm Beach, Duval, and Pinellas) [26].

LAI CAB/RPV initiation was associated with care engagement and viral suppression in the preceding year, and with being on an NNRTI-containing regimen prior to initiation. Adherence to the injection schedule is imperative for ensuring that circulating drug levels remain high enough to suppress viral replication and prevent resistance from occurring. All LAI CAB/RPV recipients achieved viral suppression before the initiation of this treatment option. This rate is higher than the 86% found in a prior analysis [33] and aligned with CAB/RPV prescribing guidelines [40]. However, it also indicates that individuals who had struggled to achieve viral suppression with oral daily pills and would be most likely to benefit from an alternative treatment modality, were not benefiting from the LAI CAB/RPV at the time of this study.

Additionally, individuals with no documented visits in the preceding year were less likely to initiate LAI CAB/RPV. These individuals may have been new to the healthcare system and may not be established enough for providers to feel comfortable offering LAI CAB/RPV, alternatively, they may have dropped out of care or moved out of the system and be receiving care, potentially including LAI CAB/RPV, elsewhere. However, we observed a small proportion of people who initiated LAI CAB/RPV also do not have records of HIV care engagement in the preceding year, indicating they were not entirely excluded. Those who initiated LAI CAB/RPV were more likely to have been on an NNRTI-containing regimen than those who continued on oral ART. NNRTI resistance has estimated prevalence of 12% and there are several NNRTI resistance mutations that impact RPV, leading to concerns among providers prescribing LAI CAB/RPV, especially in cases where resistance testing is impractical [40, 41]. Therefore, providers may be more comfortable prescribing LAI CAB/RPV to someone on a successful NNRTI-containing regimen, as those patients are unlikely to have NNRTI resistance [40, 41].

PWH with documented alcohol use disorders were less likely to initiate LAI CAB/RPV, and no statistically significant differences were found among those with other documented substance use disorders. While alcohol use alone is not a contraindication for LAI CAB/RPV, those with alcohol use disorders are more likely to have indicators of liver disease which may make prescribers leery of prescribing LAI CAB/RPV when hepatotoxicity has been documented in some cases [13, 40]. Additionally, heavy alcohol use is associated with lower care engagement, and patients with non-engagement with care are generally not seen as good candidates for LAI CAB/RPV. However, these findings may not reflect a true causal association since someone may have a lifetime diagnosis of alcohol use disorder but may not currently meet those criteria, so future studies could explore this directly.

We included both the 2-month and monthly dosing regimens of CAB/RPV [42] and found the median time between injections for both fell within the 30-day window. Additionally, our analysis showed that 71.2% of injections occurred within 37 days, which is lower than the proportion reported in a previous EHR study, where 90% of injections were within 37 days [33]. However, we also observed that many LAI CAB/RPV initiators had at least one gap longer than 67 days. This finding was not necessarily an indication of suboptimal adherence to LAI CAB/RPV. We identified LAI CAB/RPV records using prescription and dispensing data. Therefore, the administration date of LAI CAB/RPV may have lagged behind the prescription date, especially during the early implementation phase. This phase often required prior authorization and handling of insurance denials, and faced specialty pharmacy issues, causing long wait times between prescription and initiation [43]. It did not appear that oral bridging was used as the oral pills observed during the LAI period were not oral CAB/RPV. Despite the lack of evidence indicating optimal LAI CAB/RPV adherence, only one case of virologic failure was observed after LAI CAB/RPV initiation. This low rate was consistent with past study [33]. Almost half of LAI recipients continued to receive oral ART after LAI initiation. Potential reasons for this may include billing issues associated with LAI CAB/RPV as a new medication, side effects, or challenges in maintaining injection visit schedules. Further research is needed to understand this use pattern.

### Limitations

This study has some limitations. Incomplete data or inaccuracies in the electronic health records may influence the study findings. We anticipate this information bias to be non-differential between LAI and oral ART recipients. To mitigate this, we created a separate category for missing or unknown data variables, such as insurance status and care engagement pattern, reducing its impact on our analyses. Additionally, medication dispensing claims were only available for Medicaid, excluding claims from private or commercial insurance, which represents a limitation of our data. Second, we used a combination of prescription and dispensing records to identify people who received LA ART and analyze their use patterns. There is a small possibility that someone received the prescription but did not undergo the medication injection. Third, viral load outcomes were only calculated for a subset of individuals with available data as some PWH were identified from claims which did not contain laboratory data. Lastly, screening for alcohol use is rarely included in HIV care and the assessments of alcohol use and other substance use disorders are subject to under-diagnosis and under-documentation in EHRs, which leads to underestimation of the true rates of these disorders in the EHR. Additionally, all substance use disorders were measured over a lifetime and may not indicate current conditions. Despite this, we still found a significant difference by alcohol use.

## Conclusion

Since the approval of LAI CAB/RPV, an increasing initiation rate of this ART option has been observed among PWH. Our study suggested that people with suboptimal care engagement and substance use disorder were not excluded from the treatment with LAI CAB/RPV. While LAI CAB/RPV is considered a complete regimen for HIV, around half of individuals continued to receive oral ART, possibly due to billing issues related to LAI CAB/RPV, personalized treatment plans, concurrent Hepatitis B treatment, or bridging treatment. Consistent with the current prescribing guideline, all people achieved viral suppression before the initiation of LAI CAB/RPV. Despite limited evidence for optimal LAI CAB/RPV adherence, virologic failure remains rare among LAI CAB/RPV recipients.

**Table 1:** Comparison of demographics, social determinants of health and clinical characteristics between LA ART and oral ART recipients

	LA ART recipients	Oral ART recipients	*P*-value
Total	233	22,611	
Sex			
Female	132 (56.7%)	9717 (43%)	< 0.0001
Male	101 (43.3%)	12,891 (57%)	
Age group			
18–29	18 (7.7%)	2050 (9.1%)	0.0142
30–44	105 (45.1%)	6810 (30.1%)	
45–64	102 (43.8%)	12,366 (54.7%)	
>=65	8 (3.4%)	1385 (6.1%)	
Race/Ethnicity			
Hispanic	46 (19.7%)	3412 (15.1%)	0.2487
Non-Hispanic Black	119 (51.1%)	12,180 (53.9%)	
Non-Hispanic White	49 (21%)	4720 (20.9%)	
Non-Hispanic other	2 (0.9%)	674 (3%)	
Unknown	17 (7.3%)	1625 (7.2%)	
Insurance			
Medicaid	182 (78.1%)	14,216 (62.9%)	< 0.0001
Medicare	15 (6.4%)	1270 (5.6%)	
Private	11 (4.7%)	910 (4%)	
no-insurance	1 (0.4%)	73 (0.3%)	
Other government	1 (0.4%)	334 (1.5%)	
Unspecified other/unknown	22 (9.8%)	5794 (25.6%)	
HIV care engagement the year before index date			
Yes	201 (86.3%)	11,172 (49.4%)	< 0.0001
No	19 (8.2%)	3030 (13.4%)	
No data	13 (5.6%)	8409 (37.2%)	
Being virally suppressed the year before index date			
Yes	50 (100%)	893 (75.7%)	< 0.0001
No	0 (0%)	287 (24.3%)	
ART characteristics the year before index date*			
ART classes			
Any NNRTIs	52 (25.4)	1850 (14.6)	0.0006
Any NRTIs	178 (86.8)	11,785 (93.0)	< 0.0001
Any PIs	36 (17.6)	2777 (21.9)	0.5603
Any INSTIs	169 (82.4)	10,239 (80.8)	0.1340
ART patterns (5 mutually exclusive categories)			0.0070
NRTI + INSTI	121 (59.0)	7979 (63.0)	
NRTI + PI	12 (5.9)	1155 (9.1)	
NRTI + INSTI + PI	9 (4.4)	913 (7.2)	
NRTI + NNRTI	20 (9.8)	867 (6.8)	
Other regimens	43 (21.0)	1754 (13.9)	
Individuals at health risk due to socioeconomic and psychosocial factors, lifetime	15 (6.4%)	1559 (6.9%)	0.7840
Clinical outcomes			
Tobacco use disorder, lifetime	102 (43.8%)	10,234 (45.3%)	0.6506
Alcohol use disorder, lifetime	34 (14.6%)	4487 (19.8%)	0.0453
Cannabis use disorder, lifetime	44 (18.9%)	4304 (19%)	0.9535
Cocaine use disorder, lifetime	32 (13.7%)	3845 (17%)	0.1857
Opioid use disorder, lifetime	20 (8.6%)	2269 (10%)	0.4630
Amphetamine use disorder, lifetime	7 (3%)	1420 (6.3%)	0.0398
Sedative use disorder, lifetime	5 (2.1%)	699 (3.1%)	0.4061
CCI score, lifetime			
No comorbidity (CCI = 0)	60 (25.8%)	6473 (28.6%)	0.9453
Mild comorbidity (CCI = 1 or 2)	83 (35.6%)	7007 (31%)	
Moderate comorbidity (CCI = 3 or 4)	38 (16.3%)	3716 (16.4%)	
Severe comorbidity (CCI > = 5)	52 (22.3%)	5415 (23.9%)	

* Among people who had ART prescription or dispense records the year before index date (*n* = 12,859 total; *n* = 191 among LA ART recipients; *n* = 12,668 among oral ART recipients)

**Table 2:** Initiation, use pattern, geo-locations, and treatment outcome observed among LAI CAB/RPV recipients, *n* = 233

Total	233, *n*	%	95%CI (%)
Number of LAI CAB/RPV records			
1	74	31.7%	(28.1%, 35.3%)
2 to 4	119	51.1%	(47.2%, 55.0%)
5 to 9	31	13.3%	(10.6%, 16.0%)
10+	9	3.9%	(2.4%, 5.4%)
HIV treatment after initiation of LAI CAB/RPV			
Only received LAI CAB/RPV	126	54.1%	(50.2%, 58.0%)
Concurrently received other oral ART	102	43.8.%	(39.9%, 47.7%)
Continuation with oral ART	5	2.2%	(1.1%, 3.3%)
Average time gap in days between LAI injections (among people with 2 + records)	50.7	SD 45.3	
Median time gap in days between LAI injections (among people with 2 + records)	28	--	
Having 1 + time gap between LAI injections greater than 67 days (among people with 2 + records)			
No	91	57.2%	(52.5%, 61.9%)
Yes	68	42.8%	(38.1%, 47.5%)
Having virologic failure after LAI initiation (defined as having VL > 200 copies/ml; among people with viral load test results after LAI initiation)			
No virologic failure	52	98.1%	(95.9%, 100%)
Virologic failure after LAI initiation	1	1.9%	(0%, 4.1%)
First-three digits of ZIP code			
322 (Duval areas)	51	22.0%	(18.8%, 25.2%)
331 (Miami-Dade areas)	42	18.1%	(15.1%, 21.1%)
330 (Miami-Dade areas)	20	8.6%	(6.4%, 10.8%)
334 (Palm Beach areas)	15	6.5%	(4.6%, 8.4%)
337 (Pinellas areas)	13	5.6%	(3.8%, 7.4%)
Other ZIP codes	92	39.5%	(35.7%, 43.3%)

## Electronic Supplementary Material

Below is the link to the electronic supplementary material.

Supplementary Material 1

## Data Availability

No datasets were generated or analysed during the current study.
