# Supplementary material for: Receipt of long-acting injectable antiretroviral therapy among people with HIV in Southern US states: an assessment using electronic health records and claims data
Source: AIDS Res Ther. 2025 Feb 1;22:9. doi: 10.1186/s12981-024-00690-9 (PMC11787751; doi:10.1186/s12981-024-00690-9)
Supplement: Supplementary file 1 — Supplementary Material 1 [file 12981_2024_690_MOESM1_ESM.docx]

Appendix Table. ICD 9 and 10 codes used for clinical characteristics

|  | ICD-9 | ICD-10 |
| --- | --- | --- |
| Tabacco use disorder | 305.1 | F17 |
| Alcohol use disorder | 305.0, 303, 305.9 | F10, 291 |
| Cannabis use disorder | 305.2, 304.3 | F12 |
| Cocaine use disorder | 305.6, 304.2 | F14 |
| Opioid use disorder | 305.5, 304.0, 304.7 | F11 |
| Sedative use disorder | 305.4, 305.8, 304.1 | F13 |
| Amphetamine use disorder | 305.7, 304.4 | F15 |
| **Chronic conditions in the** **Charlson comorbidity index** |  |  |
| Myocardial infarction | 410, 412 | I21, I22, I25.2 |
| Congestive heart failure | 428, 398.91, 402.01, 402.11, 402.91, 404.01, 404.03, 404.11, 404.13, 404.91,404.93, 425.4, 425.5, 425.6, 425.7, 425.8, 425.9 | I09.9, I11.0, I13.0, I13.2, I25.5, I42.0, I42.5, I42.6, I42.7, I42.8, I42.9, I43, I50, I29.0 |
| Peripheral vascular disease | 93.0, 437.3, 440, 441, 443, 447.1, 557.1, 557.9, V43.4 | I70, I71, I73.1, I73.8, I73.9, I77.1, I79.0, I79.2, K55.1, K55.8, K55.9, Z95.8, Z95.9 |
| Cerebrovascular disease | 362.34, 430, 431, 432, 433, 434, 435, 436, 437, 438 | G45, G46, H34.0, I60, I61, I62, I63, I64, I65, I66, I67, I68, I69 |
| Dementia | 290, 294.1, 331.2 | F00, F01, F02, F03, F05.1, G30, G31.1 |
| Chronic pulmonary disease | 416.8, 416.9, 490, 491, 492, 493, 494, 495, 496, 497, 498, 499, 500, 501, 502, 503, 504, 505, 506.4, 508.1, 508.8 | I27.8, I27.9, J40, J41, J42, J43, J44, J45, J46, J47, J60, J61, J62, J63, J64, J65, J66, J67, J68.4, J70.1, J70.3 |
| Rheumatic disease | 446.5, 710.0, 710.1, 710.2, 710.3, 710.4, 714.0, 714.1 , 714.2, 714.8, 725 | M05, M06, M31.5, M32, M33, M34, M35.1, M35.3, M36.0 |
| Peptic ulcer | 531, 532, 533, 534 | K25, K26, K26, K27, K28 |
| Mild liver disease | 70.22, 70.23, 70.32, 70.33, 70.44, 70.55, 70.6, 70.9, 570, 571, 573.3, 573.4, 573.8, 573.9, V42.7 | B18, K70.0, K70.1, K70.2, K70.3, K70.9, K71.3, K71.4, K71.5, K71.7, K73, K74, K76.0, K76.2, K76.3, K76.4, K76.8, K78.9, Z94.4 |
| Diabetes without chronic complication | 250.0, 250.1, 250.2, 250.3, 250.8, 250.9 | E10.0, E10.1, E10.6, E10.8, E10.9, E11.0, E11.1, E11.6, E11.8, E11.9, E12.0, E12.1, E12.6, E12.8, E12.9, E13.0, E13.1, E13.6, E13.8, E13.9, E14.0, E14.1, E14.6, E14.8, E14.9 |
| Diabetes with chronic complication | 250.4, 250.5, 250.6, 250.7 | E10.2, E10.3, E10.4, E10.5, E10.7, E11.2, E11.3, E11.4, E11.5, E11.7, E12.2, E12.3, E12.4, E12.5, E12.7, E13.2, E13.3, E13.4, E13.5, E13.7, E14.2, E14.3, E14.4, E14.5, E14.7 |
| Hemiplegia or paraplegia | 334.1, 342, 343, 344.0, 344.6, 344.9 | G04.1, G11.4, G80.1, G80.2, G81, G82, G83.0, G83.1, G83.2, G83.3, G83.4, G83.9 |
| Renal disease | 403.01, 403.11, 403.91, 404.02, 404.03, 404.12, 404.13, 404.13, 404.92,4 04.93, 582, 583.0, 583.1, 583.2, 583.3, 583.4, 583.5, 583.6, 583.7, 585, 586, 588, V42.0, V45.1, V56 | I12.0.4, I13.1, N03.2, N03.3, N03.4, N03.5, N03.6, N03.7, N05.2, N05.3, N05.4 , N05.5 , N05.6 , N05.7, N18, N19, N25, N49.0, N49.1, N49.2, Z94.0, Z99.2 |
| Malignant tumors | 140, 141, 142, 143, 144, 145, 146, 147, 148, 149,150, 151, 152, 153, 154, 155, 156, 157, 158, 159, 160, 161, 162, 163, 164, 165, 166, 167, 168, 169, 170, 171, 172, 174, 175, 176, 177, 178, 179, 180, 181, 182, 183, 184, 185, 186, 187, 188, 189, 190, 191, 192, 193, 194, 195, 200, 201, 202, 203, 204, 205, 206, 207, 208, | C00, C01, C02, C03, C04, C05, C06, C07, C08, C09, C10, C11, C12, C13, C14, C15, C16, C17, C18, C19, C20, C21, C22, C23, C24, C25, C26, C30, C31, C32, C33, C34, C37, C38, C39, C40, C41, C43, C45, C46, C47, C48, C49, C50, C51, C52, C53, C54, C55, C56, C57, C58, C60, C61, C62, C63, C64, C65, C66, C67, C68, C69, C70, C71, C72, C73, C74, C75, C76, C81, C82, C83, C84, C85, C88, C90, C91, C92, C93, C94, C95, C96, C97 |
| Moderate or severe liver disease | 456.0, 456.1, 456.2, 572.2, 572.3, 572.4, 572.5, 572.6, 572.7, 572.8 | I85.0, I85.9, I86.4, I98.2, K70.4, K71.1, K72.1, K72.9, K76.5, K76.6, K76.7 |
| Metastatic tumor | 196, 197, 198, 199 | C77, C78, C79, C80 |
